# Evaluation of Double-Faced Tubularized Preputial Flap versus Duckett's Procedure for Repair of Penoscrotal Hypospadias with Significant Penile Curvature: A Comparative Study

**DOI:** 10.1155/2022/6996933

**Published:** 2022-09-21

**Authors:** Mohamed Shahin, Mohamed Abdalrazek, Mohamed Abdelmaboud, Ibrahim Mahmoud Elsayaad, Muhammad Abdelhafez Mahmoud, Mahmoud Abdelhady Mousa, Ahmed Elshamy, Omar Alsamahy, Mohamed Rehan, Sayed Elhady, Ibrahim Gamaan

**Affiliations:** ^1^Pediatric Surgery Unit-Department of Surgery, Al-Azhar University Hospital, New Damietta, Egypt; ^2^Pediatric Surgery Department, Al-Azhar University Hospitals, Cairo, Egypt; ^3^Pediatric Surgery Unit-Department of Surgery, Al-Azhar University Hospital, Assuit, Egypt; ^4^Pediatric Surgery Department, Al-Azhar University Girls, Cairo, Egypt; ^5^Urology Department, Al-Azhar University Hospital, New Damietta, Egypt

## Abstract

**Background:**

Proximal hypospadias, with significant curvature, is one of the most challenging anomalies. Great diversity and a large number of procedures described over the last 4 decades confirmed the fact that no single procedure has been universally accepted or successful. So, the aim of this study is to evaluate double-faced tubularized preputial flap (DFPF) versus transverse tubularized inner preputial flap (Duckett's procedure) as regards surgical outcomes, complications rate, and cosmetic results for repair of penoscrotal hypospadias with chordee. *Patients and Methods*. This was a prospective comparative study on 144 children with primary penoscrotal hypospadias with moderate or severe chordee, conducted at New Damietta and Assuit hospitals, Al-Azhar University, from March 2016 to March 2022. The patients were randomly divided into two equal groups; group A (*n* = 72) underwent DFPF, and group B (*n* = 72) underwent Duckett's procedure.

**Results:**

No significant difference was identified as regards demographic data. The follow-up period ranged from 20 to 66 months (mean of 28 months after DFPF and 31 months after Duckett's repair), and the complication rate was 20.1% (29 of 144 children). There were statistically significant differences between the two groups as regards the urethral stricture, penile rotation, and total complication rate. HOSE score was adopted for assessment of surgical outcomes, urine stream, and cosmetic results.

**Conclusions:**

The DFPF technique is feasible and reliable for one-stage repair of penoscrotal hypospadias with chordee and can be considered as a good option as it ensures better surgical and cosmetic outcomes with lower incidence of complications.

## 1. Introduction

Penoscrotal hypospadias is one of the most challenging penile anomalies. Great diversity and large number of repair procedures were described over the last 4 decades. Some pediatric surgeons utilized two-stage repair procedures to minimize complications such as fistula, stricture, and recurrence [1]. On the contrary, other surgeons adopted one-stage repair because of its advantages such as utilizing nonscarred skin, short operative time, and short hospital stay [2].

Duckett in 1980s utilized the inner aspect of the prepuce as a vascularized neo-urethral tube [3]. Unfortunately, there were several complications developed with the conventional tubularized onlay flap procedure such as penile rotation, diverticulum formation, and high incidence of urethrocutaneous fistulae [4]. The dissection of the preputial flap from the dorsal skin has been documented to affect the vascularity of skin. So, the idea of DFPF appeared where the tube is transposed to the ventral aspect of the penile shaft with its skin coverage as one unit [5].

Herein, this study aimed to evaluate the DFPF technique for repair of primary penoscrotal hypospadias with moderate or severe curvature in comparison to conventional Duckett's technique regarding the complications rate and surgical outcomes obtained by HOSE score.

The study was registered at ClinicalTrials.gov (ID: NCT04605068 on 21/10/2020).

## 2. Patients and Methods

This is a prospective comparative study conducted at New Damietta and Assuit hospitals, Pediatric surgery departments, Al-Azhar University, from March 2016 to March 2022, on 144 male children presented with penoscrotal hypospadias with moderate or severe chordee. All of them were submitted for a closed enveloped method to be randomly selected for one of the surgical procedures. Group A included 72 patients submitted for double-faced tubularized preputial flap, and group B included 72 patients submitted for conventional tubularized preputial flap (Duckett).

Written informed consent was obtained from parents or caregivers. The study was approved by the Institutional Review Board and Ethics Committee (registration number: 00012367-16-02–002). It was performed in accordance with the 1964 Helsinki Declaration and its later amendments or comparable ethical standards.

### 2.1. Inclusion and Exclusion Criteria

All primary penoscrotal hypospadias pediatric cases with moderate-to-severe chordee were enrolled in the study, while circumcised patients, patients with other types of hypospadias (glanular and penile shaft), hypospadias with mild chordee which was corrected after penile degloving, recurrent hypospadias, and patients who were lost in follow-up were excluded from the study. Both techniques were performed by the same surgical team.

### 2.2. Surgical Techniques

After sterilization and draping, traction suture was taken in the glans; then, a circumferential incision was made 2–3 mm proximal to the coronal sulcus, deep down to Buck's fascia, extended proximally by two vertical incisions 10–15 mm apart along the urethral plate down to the native urethral meatus and then turning around it, followed by complete degloving of the penis down to the penopubic junction. Ventral penile curvature was assessed by an artificial erection test and measured by using a digital goniometer. All patients had severe chordee more than 30 degrees. So, the perispongiosum fibrous bands were excised, followed by transection and dissection of the urethral plate to release any proximal tethering bands. If still there was significant residual chordee, 3 ventral corporotomy incisions without grafting were performed to fully straighten the penis (one made at the point of maximum bending, second∼10 mm proximal and third∼10 mm distal to it). Glanular wings were designed.

**Figure 1:**
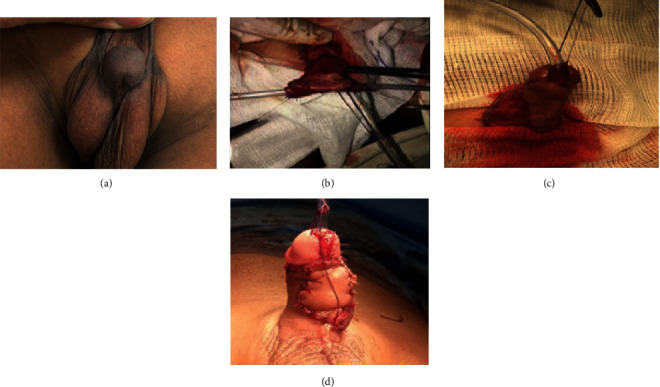
Steps of the double-faced preputial flap (DFPF) technique. (a) Penoscrotal hypospadias. (b) Preputial flap is tubularized over Nelaton's catheter. (c) Pedicle of the flap completely dissected off dorsal shaft skin and corpora down to the penopubic angle. The neo-urethral tube with still attached outer preputial layer of the flap is transposed to the ventral aspect of the penis. (d) Outer preputial layer of the flap is trimmed and sutured to the remaining dorsal shaft skin.

**Figure 2:**
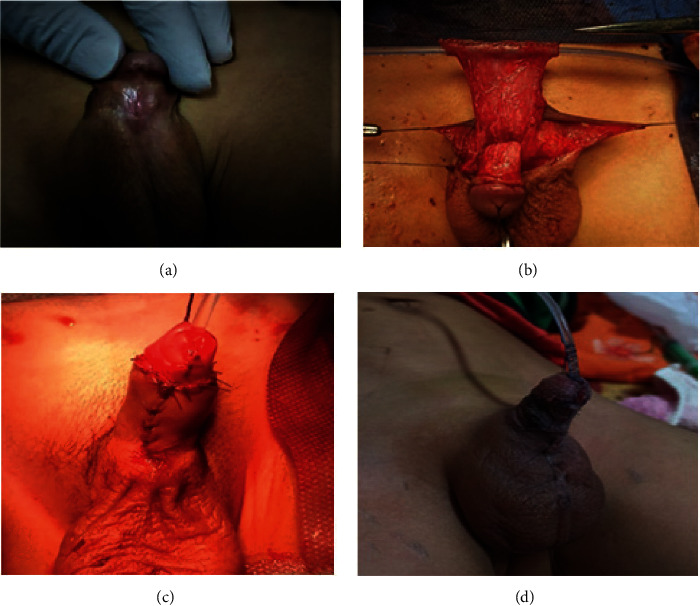
Steps of the transverse tubularized preputial island flap (Duckett's) technique. (a) Penoscrotal hypospadias. (b) Inner preputial flap is tubularized over Nelaton's catheter. (c) Neo-urethral tube is transposed to the ventral aspect of the penis with closure of skin. (d) Side view after repair was completed.

The length and width of the required preputial flap was measured and outlined. In group A, the flap was fashioned by a transverse incision at the junction of the outer preputial skin layer and dorsal penile shaft skin. The double-face flap with its vascular pedicle was dissected down to the penopubic angle. A transverse incision between the 2 faces of the flap was performed. Tubularization of the inner preputial layer was carried out over an 8 French Nelaton catheter using 6/0 vicryl sutures in the subcuticular way followed by the second layer of interrupted suture (Figures 1(a) and 1(b)). In group B, a rectangular flap was created from the inner preputial mucosal layer with its vascular pedicle dissected down to the penopubic angle, separated from outer preputial layer and dorsal penile skin. Then, the flap was tubularized in the same way of group A (Figures 2(a) and 2(b)).

In both groups, the neo-urethral tube was transposed to the ventral aspect of the penis using the button-hole technique (Figure 1(c)). The neo-urethra was fixed to the corpora and glans, using 2–3 interrupted 6/0 vicryl sutures. The suture line of neo-urethra was oriented dorsally. The native meatus was spatulated to provide wide oblique proximal anastomosis with the neo-urethral tube using interrupted 6/0 vicryl sutures. Then, the proximal anastomosis was covered and secured by scrotal dartos fascia. The neo-meatus was made oval and wide enough and wrapped by the glans. Glanuloplasty was performed by 5/0 vicryl sutures in 2 layers (interrupted then subcuticular).

In group A, preputial skin still attached to the neo-urethral tube was outlined and sutured bilaterally with the remaining penile shaft skin by 5/0 vicryl suture (Figure 1(d)). In group B, ventral skin closure was achieved using the Bayers flap from the remaining preputial and penile skin with midline closure (Figures 2(c) and 2(d)).

The catheter was fixed to the glans by 4/0 vicryl suture. Dressing was accomplished using Vaseline gauze with fusidic acid; then, sterile gauze was applied. Urine was collected using a sterile bag connected to the catheter. Immediately after operation, all patients received Diclofenac analgesic suppository.

Postoperatively, oral antibiotics, analgesics, and antispasmodics were prescribed. Dressing was removed after 4 days. The wound was left uncovered and kept moist using fusidic acid ointment. The catheter was left in place for 8–10 days postoperatively and then removed. The patients were discharged on the same instructions for care of the wound.

Follow-up at the outpatient clinic was scheduled at 1, 3, 6, and 12 months and then annually, to compare the surgical outcomes of both techniques regarding the incidence of postoperative complications in addition to the objective evaluations using Hypospadias Objective Scoring Evaluation (HOSE).

### 2.3. Statistical Analysis

Data were collected using data collecting sheets (annexes) and were analyzed using the Statistical Package for Social Sciences (SPSS) version 24.0 (IBM SPSS Statistics for Windows, IBM Corp, Armonk, NY, USA). Continuous variables were expressed as the mean ± standard deviation (SD), range, and average, while categorical variables were expressed as frequency count and percentage. Fisher's exact test was used for the comparison of frequency counts/percentage. A two-sided *P* value <0.05 was considered statistically significant.

## 3. Results

The present study included 144 children with primary penoscrotal hypospadias with chordee. Their age ranged from 1 to 4.6 years with the mean age of 2.8 years in group A (DFPF technique) and 3.1 years in group B (Duckett's technique). All patients underwent one-stage repair of penoscrotal hypospadias, using double-faced tubularized preputial flap in group A (72 cases) and transverse tubularized preputial island flap in group B (72 cases). The mean length of the neo-urethra tube was 32 mm in group A and 33 mm in group B. The follow-up period ranged between (20–62) months, and the mean was 28 months after DFPF (group A), while it ranged between (22–66) months and the mean was 31 months after Duckett's repair (group B) (Table 1). No patients were lost in the follow-up period.

The overall complication rate was 20.1% (29 of 144 children). Complications developed in 11 cases (15.3%) in group A (3 meatal stenosis, 4 penile rotations, and 4 fistulae), in comparison to 18 cases (25%) in group B (2 glans dehiscence, 2 penile rotations, 3 meatal stenosis, 3 urethral strictures, and 8 fistulae). There was a statistically significant difference between both the groups as regard to urethral stricture, penile rotation, and overall complications. A comparison between both the groups as regard the postoperative complications is shown in Table 2.

All twelve cases of urethrocutaneous fistula were successfully repaired after 6 months' interval. Two of three cases with urethral stricture responded successfully to urethral dilatations, while the third one was submitted for anastomotic urethroplasty. The two cases with glanular dehiscence were repaired successfully by redo glanuloplasty. All 6 cases of meatal stenosis were submitted for meatoplasty, while two cases of penile rotation were insignificant, and the remaining 4 cases of penile rotation were submitted for corrections.

We adopted HOSE score for postoperative objective evaluation of patients. Items of HOSE score and comparison between the groups are shown in Table 3. The postoperative HOSE score in Group A ranged between 12 and 16, and the mean was (15 ± 0.8), while the mean postoperative HOSE score in Group B was (12.9 ± 1.7), ranging between 10 and 16. The difference between the two groups regarding HOSE score was statistically significant (Table 4).

## 4. Discussion

Different procedures were described in the literature for penoscrotal hypospadias repair, predicating that none of them is ideal for these challenging cases and, therefore, the reconstructive procedure should be tailored according to the status of the urethral plate, degree of chordee, and condition of penile skin. For proximal hypospadias, preputial flap lends itself as a suitable solution for neo-urethral formation and correction of penile chordee [3–5].

Double-face tubularized preputial island flap was first described by Asopa for management of proximal hypospadias and then refined and popularized by Duckett [5].

Some studies suggest that the dissection of the preputial tube from dorsal preputial skin may affect the vascularity that leads to increased complication rate. Also, studies showed that neo-urethral tube transfer with its attached skin covering seems to achieve better results [6, 7].

Braga et al. described multiple techniques for correction of different degrees of chordee in proximal hypospadias. In their series, they reported for mild chordee, it could be corrected after just degloving of the penis. Moderate-to-severe chordee usually required additional steps. After excision of the fibrotic bands, transection and dissection of the urethral plate down to the root of the penis corrected 30–50% of severe chordee with much reduced chance of recurrence. If still there was residual chordee but less than 15 degrees, it could be further corrected by dorsal plication through the nerve-free zone [8]. However, if residual chordee is more than 15 degrees, corporotomy (or corporoplasty) was required for full straightening of the penis and augmenting the cosmetic results [9]. In this study, we came through the same way as previous studies.

Gonzalez et al. utilized double-onlay preputial flap for repair of proximal hypospadias with mild chordee. They concluded that double-face onlay flap (DFOF) combines the benefits of one-stage repair procedure beside preserving the vascularity of the flap by keeping the skin attached without separation [4]. However, in the present study, we included patients with moderate-to-severe chordee that necessitated transection and dissection of the urethral plate.

Duckett's repair has been reported to be more reliable than free tube graft operations utilizing either skin [10–13] or bladder mucosa [14].

In the study conducted by Ludwikowski and Gonzalez for utilizing total preputial flap, 4 patients developed urethrocutaneous fistula from 21 cases managed with double-faced preputial flap [15]. In another study by Singal et al. in which 92 children underwent Duckett's repair urethroplasty for proximal hypospadias, they reported that 16 (17%) patients developed 24 complications and 11 children (12%) required second surgeries [16].

In his original series, Duckett documented a complication rate ranging from 7.5% to 18% [3]. Other subsequent studies reported higher postoperative complications of one-stage Duckett's urethroplasty, varying from 8.6% to 56% [17].

Nuhoglu et al. concluded that the complication rate can be decreased by proper skin care before repair, tension-free anastomosis, fine-tissue handling, minimal use of diathermy, use of fine instruments, and wearing the optical loupe for better magnification. Also, administration of prophylactic antibiotic is mandatory if the catheter is in place [18]. In this study, we adhered to the previous principles for better outcomes.

Hayashi et al. attributed their low complication rate (7.7%) not only to 2-layer closure of the neo-urethra but also to wrapping of the proximal anastomotic site by corpus spongiosum tissue [6]. In this study, we came through the same way as Hayashi et al., as our results are comparable to their results regarding postoperative complications, especially the incidence of fistula, urethrocutaneous fistula in this study was the commonest encountered complication (4 cases in group A and 8 cases in group B). Chuang & Shieh. reported that fistula occurrence can be minimized by avoiding tissue ischemia, closure of the neo-urethra in two layers with the epithelium invaginated inwards, and additional covering of the anastomotic site with dartos fascia before skin closure [19]. Those are exactly what we adopted in this study for decreasing the incidence of fistula.

In the current study, none of the cases developed urethral diverticulum. From the authors' point of view, this may be explained by proper measurement and outlining the dimensions of the required flap to avoid redundant neo-urethra; fixation of the ventrally transposed neo-urethral tube to the corpora to avoid its laxity; and fashioning the distal neo-meatus to be oval and wide.

Daboos et al. adapted the double-faced tubularized preputial flap for repair of penoscrotal hypospadias in 80 cases from their large series about (160 cases). They have more cases of glannular dehiscence (4 cases), while in this study, there were no glannular disruptions in the double-faced group, may be due to adequate closure of glans into two layers (interrupted then subcuticular), while Daboos et al. closed the glans by 2 or 3 vicryl stitches. Although Daboos et al. have no cases of metal stenosis, in this study, we have 3 cases [20].

In this study, the mean length of the neo-urethral tube in group A was 32 mm and in group B, 33 mm, which is like the length of 33.7 mm previously documented by Hayashi et al. [6], 30 mm by Chuang and Shieh [19] and 34.6 mm by Sorber et al. [21]. It has been suggested that patients with a neo-urethra less than 3 cm in length had less significant complications than those with a neo-urethra longer than 3 cm [22]. We agree with this conclusion as 21 out of the 29 complicated patients had neo-urethra longer than 3 cm.

The preputial island tube has been reported to cause disfigurement such as ventral bulkiness and penile malrotation [23]. However, in our study we had good cosmetic results without bulking or high incidence of significant rotation of the penile shaft. This agrees with the reports of Hayashi et al. [6] as we adhered to principles of accurate measure and outline of the preputial flap to avoid redundancy. Also, we used button-hole technique in all cases of both the groups to bring either the neo-urethral tube with attached skin or tube alone in group A and group B, respectively, to the ventral aspect of the penis to avoid penile torsion.

Results after hypospadias repair can be analyzed using objective and subjective criteria. Objective criteria include evaluation of micturition by uroflowmetry, which is difficult to interpret in children as its profile is often abnormal even if reconstruction is satisfactory. Subjective criteria include cosmetic appearance and psychosocial adjustment. One of these scores is Hypospadias Objective Scoring Evaluation (HOSE) which was developed by Holland et al. [24].

By reviewing the literature, a few series had been found to adopt HOSE score for assessment of postoperative outcomes, especially after Duckett's repair. HOSE has been validated as a pediatric objective scoring system for evaluating the outcomes of hypospadias repair; as it incorporates meatal location, shape, urinary stream, the straightness of erection, and any urethral fistula. In their original series for application of HOSE score, Holland et al. used HOSE score for assessment of postoperative outcomes for different hypospadias techniques for repair of anterior and middle hypospadias. In Holland's series, HOSE assessment gave a total score ranged from 12–16 [24].

Andersson et al. adopted HOSE score in their series for assessment of urological results and patient satisfaction in adolescents after surgery for proximal hypospadias in childhood. They adopted different techniques for urethroplasty [tubularized incised plate (TIP), preputial flap as onlay, or tubularized (Duckett)]. The median HOSE score was 14 (11–16). There was no difference in HOSE between patients undergoing a TIP, onlay, or Duckett procedure [25].

A score of fourteen or more was reported by Liu et al. in their series on different techniques to infer an acceptable outcome [26]. We obtained score results like the previous studies and found that 68 cases (94%) in group A and 64 cases (88%) in group B achieved 16 points. Also, the score ranging from 10–12 was the least score for complicated cases in both the groups.

### 4.1. Limitations of the Study and Future Recommendations

Subjective assessment of erection was lacking as young children's erection is not strong enough and usually not witnessed. Urodynamics were not performed during follow-up because most patients have not yet reached the toilet training age so, they will not cooperate with the technician. Further long-term follow-up studies are strongly recommended about these repaired proximal hypospadias children as they grow from childhood to adolescence to assess long-term complications such as development of the urethral stricture, diverticulum, and recurrence of chordee.

## 5. Conclusion

Both Duckett's and DFPF techniques are good options for repair of proximal hypospadias after excision of chordee and transecting the urethral plate. Our results suggest that the DFPF repair is a superior option as it improves the vascularity of the tube and gives better results and fewer complications.

## Figures and Tables

**Table 1:** Demographic data and perioperative characteristics.

Parameter	Group A (*n* = 72)	Group B (*n* = 72)	*P* value
Age/years	2.8 (1–4.6)	3.1 (1–5)	0.36
Length of the neo-urethra tube/mm	32 (20–49)	33 (25–54)	0.51
Follow-up/months	28 (20–62)	31 (22–66)	0.08

There was no significant difference between both the groups.

**Table 2:** Postoperative complications.

Complications		Group A (*n* = 72)	Group B (*n* = 72)	*X * ^2^	*P* value
Urethrocutaneous fistula	Number	4 (5.6%)	8 (11%)	2.207	0.142
Urethral stricture	Number	0 (0%)	3 (4.2%)	5.171	0.027 ^*∗*^
Glans dehiscence	Number	0 (0%)	2 (2.8%)	3.294	0.052
Meatal stenosis	Number	3 (4.2%)	3 (4.2%)	0.000	1.000
Penile rotation	Number	4 (5.6%)	2 (2.8%)	2.085	0.048 ^*∗*^
Total	29	11 (15.3%)	18 (25%)	4.386	0.037 ^*∗*^

There was statistically significant differences between both the groups' *P* value <0.05.

**Table 3:** HOSE score comparison between the two groups.

Variable of HOSE	Score	Group A (*n* = 72)	Group B (*n* = 72)
Meatal location			
Distal glanular	4	72	70
Proximal glanular	3	0	0
Coronal	2	0	2
Penile shaft	1	0	0

Meatal shape			
Vertical slit	2	23	22
Circular	1	49	50

Urinary stream			
Single stream	2	68	64
Sprayed	1	4	8

Erection			
Straight	4	72	72
Mild angulation	3	—	—
Moderate angulation	2	—	—
Sever angulation	1	—	—

Fistula			
None	4	68	64
Single distal	3	1	2
Single proximal	2	3	6
Multiple or complex	1	—	—

**Table 4:** The difference between the total HOSE scores for both the groups was statistically significant.

Mean HOSE score	Group A (*n* = 72)	Group B (*n* = 72)	*P* value
Mean ± SD	15 ± 0.8	12.9 ± 1.7	<0.001 ^*∗*^ ^*∗*^

An independent-sample *t*-test was used; *P* value <0.001 HS.

## Data Availability

The data supporting our results are included in the article.

## Abbreviations

DFPF: Double-faced preputial flap
Fr: French
SD: Standard deviation.
